# Association between the Desire for Breast Augmentation and Instagram Engagement: A Cross-Sectional Survey among Young Polish Women

**DOI:** 10.3390/ijerph181910317

**Published:** 2021-09-30

**Authors:** Tomasz Skrzypczak, Klaudia Błachnio, Tomasz Górnicki, Justyna Kmieć, Agnieszka Ciąder, Małgorzata Biernikiewicz, Marzena Majchrowska, Małgorzata Sobieszczańska, Małgorzata Szymala-Pędzik, Dariusz Kałka

**Affiliations:** 1 Cardiosexology Students Club, Faculty of Medicine, Wroclaw Medical University, 50-368 Wrocław, Poland; t.skrzypczak.pl@gmail.com (T.S.); klaudia.blachnio1@gmail.com (K.B.); tomasz.gornicki78@gmail.com (T.G.); kmiecjustyna12345@gmail.com (J.K.); 2 IB World School 4464 in Leszno, 64-100 Leszno, Poland; agaciader@gmail.com; 3 Studio Słowa, 50-357 Wrocław, Poland; malgorzata@biernikiewicz.pl; 4 Cardiosexology Unit, Department of Physiology and Pathophysiology, Wroclaw Medical University, 50-368 Wrocław, Poland; marzen89@gmail.com; 5 Department of Geriatrics, Wroclaw Medical University, 50-369 Wrocław, Poland; malsobie100@gmail.com (M.S.); m.g.szymala@wp.pl (M.S.-P.); 6 Men’s Health Centre in Wrocław, 53-151 Wrocław, Poland

**Keywords:** breast augmentation, social media, Instagram, BREAST-Q

## Abstract

The impact of social media on the eagerness to undergo aesthetic breast surgery is unknown. We aimed to evaluate the association between Instagram engagement and the willingness to undergo breast augmentation. Women aged between 19–34 years old participated in an online survey. Of the 1560 respondents, 1226 (78.59%) met the inclusion criteria. BMI, bra type, bra cup size, education, and level of activity on Instagram increased the willingness to undergo breast augmentation (OR = 1.520, *p* = 0.020). Moreover, concurrent Snapchat use (OR = 1.348, *p* = 0.024) and the number of published posts on a respondent’s Instagram accounts (reference, *n* > 26; 0 < *n* ≤ 26; OR = 0.708, *p* = 0.009; lack of posts (*n* = 0): OR = 0.702, *p* = 0.155) were significant drivers of the respondents’ willingness. Fashion (OR = 0.730, *p* = 0.021), design/architecture (OR = 0.730, *p* = 0.022), and models (OR = 0.623, *p* = 0.004) were the searched content categories that increased the desire for breast augmentation. Positive and negative feeling scores that were triggered by Instagram content were correlated with BREAST-Q scores. We concluded that Instagram is a commonly used social network service among young women, and it may drive a desire for breast augmentation. Further analyses of Instagram preferences may help assess the willingness to undergo breast surgery, and in turn assist in tailoring marketing campaigns.

## 1. Introduction

According to the International Society of Aesthetic Plastic Surgery Global Survey Results, breast augmentation is the most popular surgical procedure worldwide. In 2018, plastic surgeons performed 1,862,506 breast augmentations, which accounted for 17.6% of the total cosmetic surgical procedures. From 2014 to 2018, a 27.6% rise in case volume was observed. In 2018, 98.8% of operations were performed on women, 53.9% of whom were 19–34-years old [1]. As the prevalence and accessibility of breast augmentations is increasing worldwide, it is becoming continuously more important to explore factors that motivate young women to undergo breast surgery.

Of all the medical specialties, plastic surgery is one most present on social media [2]. Most operations performed from head to toe have a visual component, thus synergy is not surprising [2]. Implementation of social media into plastic surgeon life has a significant impact on routine clinical practice [2,3]. Social media plays an important and growing role in plastic surgery [4], as it may potentially drive the interest in cosmetic procedures [2,5]. Many plastic surgeons feel that social media is an effective marketing tool that generates increased exposure and referrals [3]. However, some physicians experienced negative repercussions from social media involvement [3].

Viewing cosmetic surgery-related materials, spending long hours on social media platforms, and having low self-esteem are associated with an increased likelihood of considering cosmetic procedures in the future [6]. Recent studies revealed that social media influences patients’ education and decisions to undergo breast augmentation, with Instagram being the most impactful. It was demonstrated that patients who undergo breast reconstruction use social media as a source of information [7]. Although, the use of social media did not impact these patient’s expectations [7]. Up to date, the state of literature is inconclusive. The authors aimed to give new insights into this complex problem.

The increasing popularity of internet use and its growing influence on patients and surgeons is rapid. The percentage of patients reading about aesthetic surgery on social media peaked by 29.1% between 2014 and 2019. Although the percentage of surgeons that are convinced about the possibility to obtain reliable information from the internet dropped down from 61.7% in 2014 to 35.2% in 2019, it remains high. At the same time, the percentage of surgeons who are convinced that social media could create unrealistic expectations rose from 38.3% to 65.3% [8].

Facebook, YouTube, WhatsApp, Facebook Messenger, WeChat, and Instagram are among the most popular platforms worldwide [9]. In 2018, Facebook was the preferred platform for delivering comprehensive information by surgeons via live video [10]. In 2017, Facebook had the highest patient use and engagement figures, YouTube was second in the number of users, with Instagram being second in the number of engaged users [11]. In the fourth quartile of 2019, 58% of brand page followers on Instagram were females of all ages. On Facebook, women constituted 56.7% of the total audience, although there were slightly more men aged between 18–24 years old. For the first time, the whole audience on Instagram surpassed the total audience size on Facebook, with these statistics being based on the top 50 brand profiles worldwide. Additionally, there were nearly 20 times more interactions on Instagram than on Facebook. The top 50 brands published more posts on Facebook, but engagement with those posts did not reach the numbers achieved on Instagram [12].

This study aimed to examine the association between the desire for breast augmentation and Instagram engagement among 19 to 34-year-old women. To the authors’ knowledge, previously published papers focused on relationships between aesthetic surgery and social media in general. Instagram differs from other social media platforms in that Instagram’s perspective is “first photo, second text” [13]. In other words, pictures are more important than words. Similarly to other disciplines, plastic surgery accounts could take advantage of the visual power of Instagram [13]. Users spread photos and videos with hashtags and capitation’s to increase the image visibility. All Instagram users can access all shared data if the account was made public. Easy, in-app photo manipulation (morphing, filtering) potentially heighten unrealistic expectations of patients [2]. Moreover, Instagram was selected due to its balance between the number of users and their engagement rate in the platform activities [12,14]. Additionally, Instagram requires a user to be logged into their account in order to use all its features [15]. Passive, not-logged users, did not disrupt baseline engagement level. Given an increased presence of plastic surgery accounts created both by organizations and individuals on Instagram, this study attempted to investigate the impact of these on young Polish women.

## 2. Materials and Methods

### 2.1. Data Collection

An online research questionnaire was used. The survey consisted of a general part, as well as an Instagram focused section. The general component started with a demographic panel, and then three parts of the BREAST-Q Augmentation module were displayed. Participants were asked to evaluate five statements concerning their willingness to undergo breast augmentation, and then had to self-classify as an active or inactive Instagram user. The second part consisted of question blocks concerning the use of other social media platforms, the daily average time spent on Instagram, categories of searched Instagram content, the number of published posts, followed accounts, followers, Instagram preferences, and habits and feelings triggered by watched materials. The full text of the survey is in Supplementary Material Text S1. For the study, an official Polish translation and language validation of the questionnaire was conducted in adherence to the translation and Cultural Adaptation group (TCA group) principles and World Health Organization (WHO) guidelines on the translation and adaptation of instruments [16,17,18].

### 2.2. Recruitment of Participants

In order to reach an audience, the author’s Facebook and Instagram accounts were utilized. Facebook posts were published on official Polish university pages, Instagram users’ self-promotion pages, and psychology-related groups. Facebook groups and pages associated with aesthetic surgery or breast augmentation were excluded. On the author’s Instagram accounts, only public posts were published. Every social media post contained a link to the survey, information about inclusion criteria, the sensitive nature of the survey’s questions, and the possibility to leave at any time. Interested, self-classified recipients followed the link to complete the survey on the SurveyMonkey platform (SurveyMonkey Inc, San Mateo, CA, USA), which is an online web-based software used for questionnaire administration. The software allowed the survey to be filled out only once by each respondent.

### 2.3. Inclusion and Exclusion Criteria

Only Polish speaking women aged between 19–34 years old with no history of breast surgery, and who were able to complete online questionnaires, were enrolled. The second screening was applied when respondents completed the general section of the survey. The Instagram focused part of the survey was only accessible to active Instagram users. Active Instagram users were self-classified respondents who had an account that had been used in the past week. Participants who did not meet this requirement, having completed the general part of the survey, exited the questionnaire.

### 2.4. Questionnaires

The general part included three questions on the willingness to undergo aesthetic breast augmentation, and also the influence of Instagram content on the respondents’ feelings (Supplementary Material Table S1). To examine the willingness to undergo aesthetic breast augmentation, the respondents were asked to reply to five statements. Of these, the first two had positive overtones, one question expressed the unwillingness to undergo surgery, and the last two concerned the participants’ feelings regarding breast satisfaction. For each of them, one of three answers was selected by the respondents. Each option had one of three possible weights (0/1/2) assigned to it. Answer weights were summed and then divided by five. The result was an augmentation score for each of the participants. The higher the augmentation score, the greater the willingness to undergo breast augmentation.

To examine the influence of the respondent’s feelings triggered by watched Instagram content on the participant’s desire to undergo breast augmentation, positive and negative feeling scores were used. Eight emotions were displayed. Four were positive, and the rest were negative. For each of them, the respondents were asked to choose one of the five frequencies related to each emotion. Every frequency had a respective weight (1/2/3/4/5). Emotion weights were summed and then divided by four. The obtained results were positive and negative feeling scores for each of the participants. The higher the positive feeling score, the greater prevalence of positive emotions triggered by watched Instagram content. The same rule was applied to the negative feeling score.

In order to meet the criteria of data reliability, three parts of the BREAST-Q v2 augmentation module (Polish version) were used: Satisfaction with Breast Score (BSS), Psychosocial Well-Being Score (PS), Sexual Well-Being Score (SS). The BREAST-Q is rigorously developed for breast surgery and well-validated as a patient-reported outcome instrument. The augmentation module was especially designed for the evaluation of outcomes in patients seeking and undergoing breast augmentation [19,20]. In the BREAST-Q survey, the raw scores from the tables were summed and transformed into a scale ranging from 0 (worst) to 100 (best), using the Q-Score (Rasch Unidimensional Measurement Models Laboratory, Perth, Australia).

The study was performed in accordance with the Declaration of Helsinki for Human Research. According to Polish regulations included in an Act of 5 December 1996, concerning the professions of doctors and dentists [21], this research was a non-interventional, observational study and did not require the approval of the bioethics board. Data were collected and processed anonymously. This data administration model does not fall under the General Data Protection Regulation, or the mandatory European Union regulation.

### 2.5. Statistical Analysis

Data analyses were performed using IBM SPSS Statistics, version 1.0.0.1347 (IBM Corp, Armonk, NY, USA). Descriptive statistics were calculated for all variables. The Kolmogorov–Smirnov test was performed to examine the distribution of the data. Univariate analyses (Mann–Whitney U test and Kruskal–Wallis test) were executed to assess the relationships between the factors surveyed and the augmentation score.

Multinomial logistic regression analysis (MLRA) was then performed to identify significant factors for the augmentation score. MLRA enables a quantitative comparison of the separate and joint effects of putative factors [22]. As dependent variables must be categorical, continuous variables must be transformed, usually via the classification of quartiles or a value of clinical significance [23]. Thus, the dependent variable in this study (augmentation score) was dichotomized using median values as cutoff points (≤median, >median). Variables that significantly correlated with the augmentation score in univariate analyses served as independent variables in the MLRA [24]. For all of these variables, odds ratios (OR) and 95% confidence intervals (CI) were calculated. *p* < 0.005 was considered to be statistically significant.

To examine the reliability of the generated scores, Pearson’s R correlations were calculated. Correlation coefficients with 95% CI and *p* values between augmentation, positive and negative feeling scores and BSS, PS, SS were calculated.

## 3. Results

We received 1560 responses; 1226 (78.59%) respondents answered all the questions and met the inclusion criteria. Of these, 176 (14.4%) people did not have an Instagram account. The mean age of the qualified respondents was 22 ± 3 years (range 19–34). All were Polish and did not have children. The majority had a monthly personal income of less than 1500PLN (375USD; 870/1226; 70.6%), lived in cities inhabited by 500 thousand to one million people (608/1226; 49.6%), and did not have any chronic health conditions (773/1226; 63%). Table S2a,b show the participants’ demographic characteristics, BREAST-Q scores, and augmentation scores. The total mean augmentation score of the 1226 respondents was 0.69 ± 0.59; median 0.60.

The augmentation scores correlated significantly (*p* < 0.001 for all scales) with the BREAST Q scores with the following coefficients: R = −0.522 (95% CI, −0.480 to −0.561) for PS; R = −0.488 (95% CI, −0.444 to −0.529) for SS; and R = −0.660 (95% CI, −0.628 to −0.691) for BSS. In MLRA, the augmentation score was dichotomized using the median value (0.60) as a cut-off point [22].

When analyzing the augmentation scores and demographic variables, significant correlations between the augmentation scores and BMI, bra type, bra cup size (all *p* < 0.001), education (*p* = 0.024), and possession of an active Instagram account (*p* = 0.007) were found. The details are shown in Table 1.

The likelihood ratio chi-square test indicated that the chi-square value of the MLRA model for the augmentation scores was 147.670, and the *p* < 0.001. MLRA showed that the demographic factors associated with willingness to undergo breast augmentation were BMI (reference, 18.5–25.0; >25.0: OR = 0.483, *p <* 0.001; <18.5: OR = 0.934, *p =* 0.709), bra type (reference, sporty; push-up: OR = 0.224, *p <* 0.001; half-cup: OR = 0.656, *p =* 0.178; full-cup: OR = 0.761, *p =* 0.029; other: OR = 0.761, *p =* 0.447; balconette: OR = 0.632, *p =* 0.274), bra cup size (reference, D size; C size: OR = 0.796, *p =* 0.284; B size: OR = 0.572, *p =* 0.006; A size: OR = 0.195, *p <* 0.001; > D size: OR = 0.597, *p =* 0.022) and active Instagram account (OR = 1.520, *p =* 0.020). See Table 2 for further details.

For 1050 active Instagram account holders, the mean number of published posts was 77,432 ± 240 (median = 26), mean number of followers was 806,634 ± 7195 (median = 279), and the mean number of followed users was 364,106 ± 337 (median = 300). The univariate analysis presented in Table 3 showed significant correlations between augmentation scores and Snapchat (*p* < 0.001), TikTok (*p =* 0.025), number of posts published on a respondent’s Instagram accounts (*p =* 0.003).

**Table 3:** Univariate analysis of associations between augmentation scores and other social media platforms use, daily time spent on Instagram and respondents’ activity on this platform (*n* = 1050).

Variable		Augmentation Score		Variable		Augmentation Score	
		Mean ± SD	*p*			Mean ± SD	*p*
Daily Time Spending on Instagram				TikTok			
	<0.5 h	0.667 ± 0.57	0.855		Yes	0.809 ± 0.64	0.025
	0.5–1 h	0.706 ± 0.60			No	0.687 ± 0.59	
	1–2 h	0.729 ± 0.63		Other			
	>2 h	0.712 ± 0.60			Yes	0.804 ± 0.53	0.103
Facebook					No	0.701 ± 0.60	
	Yes	0.707 ± 0.60	0.837	Number of published posts			
	No	0.677 ± 0.64			0	0.752 ± 0.55	0.003
YouTube					0 < x ≤ 26	0.771 ± 0.62	
	Yes	0.701 ± 0.60	0.312		x > 26	0.644 ± 0.58	
	No	0.775 ± 0.62		Number of followers			
Twitter					0	0.686 ± 0.65	0.421
	Yes	0.733 ± 0.58	0.473		0 < x ≤ 279	0.674 ± 0.57	
	No	0.703 ± 0.60			x > 279	0.739 ± 0.63	
Snapchat				Number of followed accounts			
	Yes	0.789 ± 0.63	<0.001		0	0.480 ± 0.66	0.635
	No	0.639 ± 0.57			0 < x ≤ 300	0.704 ± 0.59	
Pintrest					x > 300	0.712 ± 0.61	
	Yes	0.725 ± 0.62	0.658				
	No	0.699 ± 0.59					

Likelihood ratio chi-square test indicated that the chi-square value of MLRA model for augmentation scores was 17.967 and the *p =* 0.001. MLRA showed that Snapchat use (OR = 1.348, *p =* 0.024) and number of published posts on respondent’s Instagram accounts (reference, *n* > 26; 0 < *n* ≤ 26: OR = 0.708, *p =* 0.009; lack of posts (*n* = 0): OR = 0.702, *p =* 0.155) correlates with augmentation score. The detailed results of MLRA are presented in Table 4.

**Table 4:** Multinomial logistic regression analysis of association between other social media platforms use, number of published posts and augmentation score.

Variable		*p*	OR	95% CI
Snapchat		0.024		
	NO	0.024	1.348	1.041–1.747
	YES	.	.	.
TikTok		0.335		
	NO	0.335	1.184	0.840–1.670
	YES	.	.	.
Number of published posts		0.023		
	0	0.155	0.702	0.431–1.144
	0 < x ≤ 26	0.009	0.708	0.547–0.917
	x > 26	.	.	.

Likelihood ratio chi-square test indicated that the χ^2^ value of MLRA model for augmentation scores was 17.967 and the *p* < 0.001. Augmentation score was dependent variable, dichotomized (≤median, >median). Cut-off point (median) was 0.60.

To examine correlations between searched content categories on Instagram and the augmentation score, univariate analysis was performed (Table 5). Fashion, models (all *p* < 0.001), design/architecture (*p =* 0.018), celebrities (*p =* 0.010), wildlife (*p =* 0.003) were categories that had strong relationships with augmentation scores. Results of MRLA analysis are presented in Table 6. Likelihood ratio chi-square test indicated that the chi-square value of the MLRA model for augmentation scores was 31.660 and the *p* < 0.001. MLRA showed that fashion (OR = 0.730, *p =* 0.021), design/architecture (OR = 0.730, *p =* 0.022), models (OR = 0.623, *p =* 0.004) were searched content categories corelated with willingness to undergo breast augmentation.

Table 7 shows correlations between Instagram use preferences and habits and augmentation scores, revealed by univariate analysis. A significant relationship between the necessity of wearing make-up for the perfect Instagram picture and augmentation scores was found (*p* < 0.001). Readiness to publish photos on Instagram with respondent’s face fully visible was associated with willingness to undergo surgery (*p =* 0.002). The likelihood ratio chi-square test indicated that the chi-square value of the MLRA model for augmentation scores was 45.389 and the *p* < 0.001. MLRA showed the necessity of wearing make-up for a perfect Instagram picture (reference, definitely agree; somewhat agree: OR = 1.721, *p =* 0.001; disagree: OR = 2.498, *p* < 0.001), readiness to publish photos on Instagram with respondent’s face fully visible (reference, definitely agree; somewhat agree: OR = 0.784, *p =* 0.084; disagree: OR = 0.582, *p =* 0.002) coexisted with the desire to undergo breast augmentation. See Table 8 for further details.

Positive and negative feelings scores significantly correlated with BSS, PS, and SS with *p* < 0.001 for each comparison. Positive feelings scores strongly correlated with PS [R = 0.730 (95% CI, 0.757–0.700)], SS [R = 0.672 (0.704–0.638)] and BSS [R = 0.568 (0.608–0.526)]. Correlations were also found between negative feeling scores and PS [R = −0.671 (95% CI, −0.636–−0.703)], SS [R = −0.587 (−0.545–−0.625)] and BSS [R = −0.547 (95% CI, −0.503–−0.588)]. Similarly, significant correlations were found between positive and negative feeling scores and augmentation scores. Augmentation scores were moderately correlated with positive and negative feeling scores [R = −0.380 (95% CI, −0.327–−0.431) and R = 0.375 (0.426–0.322), respectively], all *p* < 0.001.

## 4. Discussion

Instagram is common among young Polish women (19–34 years old) with 67% of them using the platform. Having an active Instagram account increased the willingness to undergo breast augmentation. In addition, engagement in the activity on social media, as measured using Snapchat parallelly, and a number of published Instagram posts were the drivers for breast augmentation. Searched content categories on Instagram was important as well. Fashion, models, design/architecture, celebrities, and wildlife were categories that had strong relationships with augmentation scores. Wearing make-up for the perfect Instagram picture and readiness to publish photos on Instagram with respondent’s face fully visible were both significantly associated with willingness to undergo surgery. Finally, positive, and negative feelings scores triggered by watched Instagram content correlated with BREAST-Q components scores.

This study is a cross-sectional study with self-reported data, which might introduce response bias due to social desirability or selective memory (recall bias) [6]. Moreover, an online survey carries specific limitations with it. Such a tool does not allow for shaping the target population or selection of a more specific study group. Furthermore, it might attract more women who are interested in breast augmentation. For this reason, the results should be generalized carefully.

A specified attention to click question was not applied. In this study, we used a validated BREAST-Q questionnaire; however, augmentation score questions were not validated, so that the augmentation score cannot be entirely trustworthy. Despite those limitations, the present study constitutes an important step in determining the association between Instagram exposure and young women’s desire for breast augmentation. This study had a larger sample than previous online questionnaire studies [10,25] and utilized a validated research instrument. In contrast to other articles [11,25], only one procedure and highly engaging social media platform were investigated in detail. These provide a more specific insight for interested plastic surgeons.

The main discovery of the present study was that the active Instagram account used within the past week was correlated with the respondent’s willingness to undergo breast augmentation, similarly to BMI, bra cup size and bra type. On the contrary, age, education, marital status, monthly personal income, inhabited city population, and long-term health condition were not significant. This coincided with findings reported by Tiggemann et al., who claimed that the real post has the potential to bolster women’s body satisfaction [26]. Similarly, Javo et al., using the multiple regression analysis, found that age and marital status did not correlate with interest in breast augmentation, in contrast to low education level, which was a strong predictor of interest in breast augmentation [27]. Furthermore, Frederick et al. found that BMI had an impact on willingness to undergo breast augmentation [28]. In another prospective study, marital status was not a motivating factor. Yet, women were extremely motivated to have breast implant surgery to make themselves feel better about their physical appearance [29].

We found that among active Instagram users, a number of posts published on a respondent’s account was associated with willingness to undergo breast augmentation. In opposition to a number of followers and followed users, which did not indicate such correlation. The number of posts higher than 0 and lower than 26 were associated with the greatest mean augmentation score. Snapchat was the only platform that, when used parallelly, had statistically significant correlation. These results confirmed concerns raised by Ramphul and Mejias [30]. Apart from active Instagram use, collateral Snapchat use was associated with greater interest in surgery. Secondary, Facebook, YouTube, Twitter, Pinterest and TikTok usage were not statistically significant. Daily average time spent on Instagram did not increase interest in surgery. In contrast, more than five hours spent on social media platforms was associated with greater interest to undergo cosmetic procedures in the future [6]. This conclusion could be misleading because it was based only on univariate analysis. Walker et al., in their prospective study, found the opposite result to those from the present study. A significant amount of time on social media and following many accounts affected young women’s desire for cosmetic surgery [31]. In contrast to the present study, the study by Walker et al. had a smaller study sample (118 in Walder’s et al. study vs. 1226 in the present study). A different methodology based on viewing images of cosmetic procedures could be another factor that led to opposite conclusions.

In the present study, positive feeling scores strongly correlated with BSS, PS, and SS of the BREAST-Q questionnaire. Similar relationships were observed for negative feelings scores. BREAST-Q scores correlated with augmentation scores. For this reason, the augmentation score might be considered a measure method of a respondent’s desire to undergo breast augmentation [32]. However, before its application in further studies, we recommend prior validation with a method published by Faye-Dumanget et al. [33].

Searching for content in the following categories: fashion, models and design/architecture, was associated with willingness to undergo breast augmentation. In contrast to friends, travelling, food, celebrities, inspiration and wildlife, which were not. Increased prevalence of negative feelings triggered by watched Instagram content correlated with growth in breast augmentation interest. The opposite was revealed for positive feelings. Complementary results were found in another study. Fardouly et al. [34] found that Instagram usage might negatively impact women’s concerns and beliefs about their appearance. Brown and Tiggemann [35] revealed that exposure to attractive celebrity and peer Instagram images could increase body dissatisfaction. Instagram images in the travel category did not have a significant correlation. Contrary to the present research, the Brown and Tiggemann [35] study sample consisted of 138 females from one university. Findings for the larger and more diverse population could be different.

The necessity of wearing make-up for the perfect Instagram picture and readiness to publish photos on Instagram with the respondent’s face fully visible were associated with breast augmentation interest, as the present study found. Other Instagram preferences, such as as publishing most of the photos on Instagram, the necessity of in-app filters use for the perfect picture, readiness to upload photos with exposed neckline, willingness to upload participant’s naked photos and professional photographer assistance were not significantly correlated with willingness to undergo breast augmentation. In a previous study by Sarwer et al., women interested in breast augmentation reported greater investment in their appearance, greater distress about their appearance in a variety of situations, and more frequent teasing about their appearance [36]. These results could explain mandatory make-up wear as a remedy for overwhelming appearance concerns.

## 5. Conclusions

Instagram is common among young women, and it may drive a desire for breast augmentation. Instagram users with a moderate number of posts seek materials about breast augmentation the most intensely. Adverts targeting women searching for fashion information, professional models’ sessions, and design and make-up may have an excellent outcome for aesthetic surgeons but a negative impact on people mental well-being. Further analyses of Instagram preferences may be helpful for the assessment of willingness to undergo breast surgery and tailoring marketing campaigns.

## Figures and Tables

**Table 1:** Univariate analysis of associations between augmentation scores and demographic data.

Variable		Augmentation Score		Variable	Augmentation Score	
		Mean ± SD	*p*		Mean ± SD	*p*
Age				Population of inhabited city		
	19–25	0.691 ± 0.60	0.662	500,000–1,000,000	0.649 ± 0.59	0.159
	26–34	0.666 ± 0.59		250,000–500,000	0.694 ± 0.62	
BMI				100,000–250,000	0.788 ± 0.64	
	<18.5	0.752 ± 0.65	<0.001	10,000–100,000	0.733 ± 0.58	
	18.5–25.0	0.651 ± 0.59		<10,000	0.741 ± 0.58	
	>25.0	0.819 ± 0.58		<1000	0.684 ± 0.60	
Educational level				Long-term health condition		
	Elementary school	0.747 ± 0.64	0.024	Yes	0.694 ± 0.60	0.769
	Secondary school	0.784 ± 0.61		No	0.685 ± 0.60	
	Undergraduate student	0.716 ± 0.59		Size of bra cup		
	Professional school student	0.624 ± 0.58		A	1.083 ± 0.61	<0.001
	Bachelor’s degree	0.677 ± 0.58		B	0.732 ± 0.61	
	Master’s degree	0.675 ± 0.63		C	0.584 ± 0.59	
	Doctor of Philosophy	0.819 ± 0.58		D	0.507 ± 0.49	
Marital status				>D	0.613 ± 0.52	
	Single	0.736 ± 0.60	0.296	Preferred bra type		
	Living with significant other	0.681 ± 0.60		Full-cup	0.638 ± 0.55	<0.001
	Married	0.605 ± 0.61		Half-cup	0.576 ± 0.533	
	Alone	0.655 ± 0.54		Push-up	1.025 ± 0.652	
	Divorced	0.400 ± 0.28		Sporty	0.479 ± 0.532	
Monthly personal income				Balconette	0.565 ± 0.57	
	<375 USD	0.686 ± 0.59	0.831	Other	0.518 ± 0.55	
	375–1125 USD	0.699 ± 0.62		Active Instagram account		
	>1125 USD	0.661 ± 0.67		Yes	0.707 ± 0.60	0.007
				No	0.580 ± 0.57	

**Table 2:** Multinomial logistic regression analysis of associations between demographic data and augmentation score.

Variable		*p*	OR	95% CI
BMI		<0.001		
	<18.5	0.709	0.934	0.651–1.339
	>25.0	0.000	0.483	0.336–0.693
	18.5–25.0	.	.	.
Educational level		0.309		
	Phd	0.676	1.394	0.293–6.630
	BSc	0.193	1.405	0.842–2.344
	MSc	0.142	1.440	0.885–2.343
	Primary school	0.217	1.599	0.759–3.368
	Professional School student	0.010	1.555	1.113–2.173
	Undergraduate student	0.092	1.373	0.950–1.985
	High School	.	.	.
Size of bra cup		<0.001		
	>D	0.022	0.597	0.384–0.929
	A	<0.001	0.195	0.117–0.326
	B	0.006	0.572	0.382–0.855
	C	0.284	0.796	0.524–1.209
	D	.	.	.
Preferred bra type		<0.001		
	balconette	0.247	0.632	0.291–1.374
	other	0.447	0.761	0.377–1.537
	full-cup	0.029	0.506	0.274–0.934
	half-cup	0.178	0.656	0.356–1.211
	push-up	<0.001	0.224	0.120–0.417
	sporty	.	.	.
Active Instagram account		0.020		
	NO	0.021	1.520	1.065–2.171
	YES	.	.	.

Likelihood ratio chi-square test indicated that the χ^2^ value of MLRA model for augmentation scores was 147.670 and the *p* < 0.001. Augmentation score was dependent variable, dichotomized (≤median, >median). Cut-off point (median) was 0.60.

**Table 5:** Univariate analysis of associations between augmentation scores and Instagram content categories (*n* = 1050).

Variable		Augmentation Score		Variable		Augmentation Score	
		Mean ± SD	*p*			Mean ± SD	*p*
Friends				Models			
	Yes	0.706 ± 0.60	0.895		Yes	0.914 ± 0.66	<0.001
	No	0.712 ± 0.63			No	0.644 ± 0.57	
Fashion				Celebrities			
	Yes	0.774 ± 0.61	<0.001		Yes	0.768 ± 0.60	0.010
	No	0.639 ± 0.58			No	0.674 ± 0.60	
Travelling				Inspirations			
	Yes	0.684 ± 0.60	0.177		Yes	0.686 ± 0.59	0.191
	No	0.730 ± 0.60			No	0.739 ± 0.61	
Food				Wildlife			
	Yes	0.691 ± 0.60	0.426		Yes	0.599 ± 0.55	0.003
	No	0.723 ± 0.61			No	0.737 ± 0.61	
Design/architecture				Other			
	Yes	0.642 ± 0.58	0.018		Yes	0.645 ± 0.58	0.066
	No	0.707 ± 0.60			No	0.726 ± 0.61	

**Table 6:** Multinomial logistic regression analysis of association between searched Instagram content categories and augmentation score.

Variable		*p*	OR	95% CI
Fashion				
	Yes	0.021	0.730	0.559–0.954
	No			
Design/architecture				
	Yes	0.022	0.730	0.557–0.956
	No			
Models				
	Yes	0.004	0.623	0.452–0.857
	No			
Celebrities				
	Yes	0.686	0.946	0.721–1.240
	No			
Wildlife				
	Yes	0.090	1.304	0.960–1.772
	No			

Likelihood ratio chi-square test indicated that the χ2 value of MLRA model for augmentation scores was 31.660 and the *p* < 0.001. Augmentation score was dependent variable, dichotomized (≤median, >median). Cut-off point (median) was 0.60.

**Table 7:** Univariate analysis of associations between augmentation scores and respondent’s Instagram habits and preferences (*n* = 1050).

Variable		Augmentation Score	
		mean ± SD	*p*
Instagram is the portal where I publish the most of my photos.			
	
	Disagree	0.759 ± 0.61	0.204
	Somewhat agree	0.707 ± 0.60	
	Definitely agree	0.681 ± 0.60	
I publish photos with use of in-app filters or other digital image enhancing techniques.			
	
	Disagree	0.678 ± 0.60	0.323
	Somewhat agree	0.696 ± 0.60	
	Definitely agree	0.744 ± 0.61	
Make-up wear is necessary for perfect Instagram photography.			
	
	Disagree	0.559 ± 0.54	<0.001
	Somewhat agree	0.708 ± 0.61	
	Definitely agree	0.894 ± 0.61	
My face is fully visible on my published photos.			
	
	Disagree	0.817 ± 0.61	0.002
	Somewhat agree	0.728 ± 0.58	
	Definitely agree	0.650 ± 0.60	
I publish photos with exposed neckline.			
	
	Disagree	0.725 ± 0.60	0.120
	Somewhat agree	0.645 ± 0.58	
	Definitely agree	0.571 ± 0.61	
I think I could publish naked photos.			
	
	Disagree	0.720 ± 0.61	0.121
	Somewhat agree	0.564 ± 0.48	
	Definitely agree	0.567 ± 0.56	
I often ask a person with experience in professional photography for help.			
	
	Disagree	0.712 ± 0.60	0.547
	Somewhat agree	0.635 ± 0.59	
	Definitely agree	0.719 ± 0.62	

**Table 8:** Multinomial logistic regression analysis of association between respondent’s Instagram habits and preferences and augmentation scores.

Variable		*p*	OR	95% CI
Make-up wear is necessary for perfect Instagram photography.				
		<0.001		
	Disagree	<0.001	2.498	1.835–3.400
	Somewhat agree	0.001	1.721	1.259–2.351
	Definitely agree			
My face is fully visible on my published photos.				
		0.008		
	Disagree	0.002	0.582	0.411–0.826
	Somewhat agree	0.084	0.784	0.595–1.033
	Definitely agree			

Likelihood ratio chi-square test indicated that the χ^2^ value of MLRA model for augmentation scores was 45.389 and the *p* < 0.001. Augmentation score was dependent variable, dichotomized (≤median, >median). Cutoff point (median) was 0.60.

## Data Availability

The data presented in this study are available in the manuscript and supplementary materials.

## Supplementary Materials

The following are available online at https://www.mdpi.com/article/10.3390/ijerph181910317/s1, Table S1: Questions on willingness to undergo breast augmentation surgery and emotions triggered by Instagram content concerning the past week. Table S2a: Demographic characteristic of respondents (*N* = 1226) Table S2b: BREAST Q and Augmentation score results. Text S1: Research survey.
