# Supplementary material for: Association between the Desire for Breast Augmentation and Instagram Engagement: A Cross-Sectional Survey among Young Polish Women
Source: Int J Environ Res Public Health. 2021 Sep 30;18(19):10317. doi: 10.3390/ijerph181910317 (PMC8508550; doi:10.3390/ijerph181910317)
Supplement: Supplementary file 1 [file ijerph-18-10317-s001.zip › ijerph-1373677-supplementary.pdf]

Supplementary Material

Title: Association between breast augmentation desire and Instagram engagement: a cross-sectional survey among young Polish women

Table S1. Questions on willingness to undergo breast augmentation surgery and emotions triggered by Instagram content concerning the past week.

**Willingness to undergo breast augmentation surgery**					
Please address the following statements		disagree		somewhat agree	definitely agree
I think the surgery would be a good experience.		0		1	2
The surgery would change my life for the better.		0		1	2
I do not feel that I need surgery to improve my breasts' look.		2		1	0
My breasts are the perfect size for me.		2		1	0
My breasts perfectly match my expectation.		2		1	0
Positive emotions	None of the time?	A little of the time?	Some of the time?	Most of the time?	All of the time?
**Concerning period of past week and watched Instagram content, how often did you feel?**					
Beautiful?	1	2	3	4	5
Admired by others?	1	2	3	4	5
Attractive?	1	2	3	4	5
Equally worth as other women?	1	2	3	4	5
Negative emotions	None of the time?	A little of the time?	Some of the time?	Most of the time?	All of the time?
**Concerning period of past week and watched Instagram content, how often did you feel?**					
Average?	1	2	3	4	5
Unpopular?	1	2	3	4	5
Ugly?	1	2	3	4	5
Worse than other women?	1	2	3	4	5

Table S2a. Demographic characteristic of respondents (N=1226).

**Variable**		n	%N	**Augmentation Score (n)**	
				**≤Median**	**>Median**
Age					
	19-25	1093	89.15	619	474
	26-34	133	10.85	71	62
BMI					
	<18.5	171	13.95	86	85
	18.5-25.0	886	72.27	527	359
	>25.0	169	13.78	77	92
Education					
	Elementary school	38	3.10	22	16
	Secondary school	248	20.23	122	126
	Undergraduate student	269	21.94	150	119
	Professional school student	463	37.77	278	185
	Bachelor's degree	91	7.42	52	39
	Master's degree	110	8.97	62	48
	Doctor of Philosophy	7	0.57	4	3
Marital status					
	Single	333	27.16	177	156
	Living with significant other	716	58.40	409	307
	Married	80	6.53	51	29
	Alone	95	7.75	51	44
	Divorced	2	0.16	2	0
Monthly personal income					
	<375 USD	870	70.96	490	380

	375-1125 USD	320	26.10	180	140
	>1125 USD	36	2.94	20	16
Population of inhabited city					
	500.000-1.000.000	608	49.59	355	253
	250.000-500.000	170	13.87	94	76
	100.000-250.000	99	8.08	50	49
	10.000-100.000	187	15.25	99	88
	<10.000	95	7.75	52	43
	<1.000	67	5.46	40	27
Long-term health condition					
	Yes	453	36.95	257	196
	No	773	63.05	433	340
Size of bra cup					
	A	151	12.32	45	106
	B	410	33.44	277	183
	C	293	23.90	185	108
	D	179	14.60	123	56
	>D	193	15.74	110	83
Bra type					
	Full-cup	365	29.77	213	152
	Half-cup	348	28.38	226	122
	Push-up	285	23.25	102	183
	Sporty	63	5.14	44	19
	Balconette	62	5.06	39	23
	Other	103	8.40	66	37
Active Instagram account					
	Yes	1050	85.64	575	475
	No	176	14.36	115	61

Table S2b. BREAST Q and Augmentation score results.

Variable	n	Mean	SD
Psychosocial Well-Being Score	1226	61.97	19.38
Sexual Well-Being Score	1226	55.8	21.35
Satisfaction with Breast Score	1226	53.7	15.77
Augmentation score	1226	0.69	0.59

Text S1. Research survey.

Research survey, overall 37 items, average time 7 min 20 sec.

General component (18 items)

Demographic section (12 items)

1.How old are you? Please enter your age.
2.What is your height? Please enter your height (in cm).
3 How much do you weigh? Please enter your weight (in kg).
4. What is your nationality?
 a) Polish
 b) Ukrainian
 c) Russian
 d) other
5.Your education level is:
 a) Elementary school
 b) Secondary school
 c) Undergraduate student
 d) Bachelor's degree
 e) Professional school student
 f) Master's degree
 g) Doctor of Philosophy
6.Your marital status? What definition does suit you the best?
 a) Alone (never married or in relationship)
 b) Living with significant other
 c) Married
 d) Divorced
 e) Widowed
 f) Other (e.g.: separated)
7.What is your monthly personal income?
 a) Less than 1500PLN(375USD)
 b) From 1500PLN to 4500PLN (375-1125USD)
 c) More than 4500 PLN(1125USD)
8.What is the population of the city you live in?
 a) Town under 1 thousand (K)
 b) Town under 10 K
 c) 10K- 100K (e.g: Legnica, Leszno)
 d) 100K-250K (e.g: Sosnowiec, Zabrze)
 e) 500K - 1mln (e.g: Warszawa, Kraków, Łódź, Poznań, Wrocław)
9.Do you suffer from chronic diseases?
 a) Yes
 b) No
10.What is bra cup size you wear on daily basis?
 a) A
 b) B
 c) C
 d) D
 e) >D
11.What type of bra do you wear most often?
 a) balconette (lowered cup, cut nearly horizontally)
 b) push up
 c) full cup

d) half-cup
e) sports bra
f) other

12.Do you have children?
a) I do not have children
b) one
c) two
d) more than two

BREAST Q section.

In this section three parts of BREAST-Q version 2.0© Augmentation Module, Polish (PL) Version: PSYCHOLOSOCIAL WELL-BEING, SEXUAL WELL-BEING, and BREAST SATISFACTION were utilized.

Examination of respondent willingness to undergo breast augmentation. (5 items)

We would like to know your opinion on the breast augmentation surgery. Please indicate to what extent you agree or disagree with following statements.

"Concerning period of past week, please address following statements"	disagree	somewhat agree	definitely agree
"I think, the surgery would be a good experience"	0	1	2
"The surgery would change my life for the better"	0	1	2
"I do not feel that I need surgery to improve my breasts' look"	2	1	0
"My breasts are the perfect size for me"	2	1	0
"My breasts perfectly match my expectation"	2	1	0

Self-classification question

Do you have an active Instagram account, that was used in the past week?

a) Yes, I used it over the past week
b) No

Instagram focused part – available only to self-classified active Instagram users. (19 items)

Apart from Instagram, what social media platforms do you use?

a) Facebook
b) YouTube
c) Twitter
d) Snapchat
e) TikTok
f) Pinterest
g) Other

How much time do you spent on Instagram, daily?

a) Less than 30min a day
b) 0,5-1h a day
c) 1-2h a day
d) More than 2h a day

On Instagram I search for content in the following categories: (multiple choice)

a) Friends
b) Fashion
c) Travelling
d) Food
e) Design/Architecture
f) Models
g) Celebrities
h) Inspiration
i) Wildlife
j) Other

Instagram habits & preferences (7 items)

We would like to get to know your preferences regarding Instagram. Please indicate to what extent you agree or disagree with following statements.

Instagram is portal where I publish the most of my photos.	disagree	somewhat agree	definitely agree
I publish photos with use in-app filters or other digital image enhancing techniques.	disagree	somewhat agree	definitely agree
Make-up wear is necessary for perfect Instagram photography.	disagree	somewhat agree	definitely agree
My face is fully visible on my published photos.	disagree	somewhat agree	definitely agree
I publish photos with exposed neckline.	disagree	somewhat agree	definitely agree
I think I could publish naked photos.	disagree	somewhat agree	definitely agree
I often ask person with experience in professional photography for help.	disagree	somewhat agree	definitely agree

How are you popular on Instagram? Please enter your account details.

a) Number of published posts
b) Number of followers

c) Number of followed accounts

<u>Feelings triggered by watched Instagram content.</u> (8items)

Concerning period of past week and watched Instagram content, how often did you feel?

	None of the time?	A little of the time?	Some of the time?	Most of the time?	All of the time?
Beautiful?	1	2	3	4	5
Admired by others?	1	2	3	4	5
Attractive?	1	2	3	4	5
Equally worth as other women?	1	2	3	4	5
Average?	1	2	3	4	5
Unpopular?	1	2	3	4	5
Ugly?	1	2	3	4	5
Worse than other women?	1	2	3	4	5
